# MotifClick: prediction of *cis*-regulatory binding sites via merging cliques

**DOI:** 10.1186/1471-2105-12-238

**Published:** 2011-06-16

**Authors:** Shaoqiang Zhang, Shan Li, Meng Niu, Phuc T Pham, Zhengchang Su

**Affiliations:** 1 Department of Bioinformatics and Genomics, Center for Bioinformatics Research, the University of North Carolina at Charlotte, 351 Bioinformatics Building, 9201 University City Blvd., Charlotte, NC 28223, USA; 2 College of Computer and Information Engineering, Tianjin Normal University, 393 Bin Shui Xi Road, Tianjin, 300387, China

## Abstract

**Background:**

Although dozens of algorithms and tools have been developed to find a set of *cis*-regulatory binding sites called a motif in a set of intergenic sequences using various approaches, most of these tools focus on identifying binding sites that are significantly different from their background sequences. However, some motifs may have a similar nucleotide distribution to that of their background sequences. Therefore, such binding sites can be missed by these tools.

**Results:**

Here, we present a graph-based polynomial-time algorithm, MotifClick, for the prediction of *cis*-regulatory binding sites, in particular, those that have a similar nucleotide distribution to that of their background sequences. To find binding sites with length *k*, we construct a graph using some 2(*k*-1)-mers in the input sequences as the vertices, and connect two vertices by an edge if the maximum number of matches of the local gapless alignments between the two 2(*k*-1)-mers is greater than a cutoff value. We identify a motif as a set of similar *k*-mers from a merged group of maximum cliques associated with some vertices.

**Conclusions:**

When evaluated on both synthetic and real datasets of prokaryotes and eukaryotes, MotifClick outperforms existing leading motif-finding tools for prediction accuracy and balancing the prediction sensitivity and specificity in general. In particular, when the distribution of nucleotides of binding sites is similar to that of their background sequences, MotifClick is more likely to identify the binding sites than the other tools.

## Background

Deciphering complex genetic regulatory networks encoded in a genome is a challenging problem in the post-genomic era [1]. Identifying *cis*-regulatory binding sites recognized by transcription factors (TF) in a genome is the first step towards this goal [2]. Since any segment of genomic sequence can be potentially a *cis*-regulatory binding site, a binding site motif (In this paper, we call a set of similar *cis*-regulatory binding sites recognized by the same TF a *motif*) can only be predicted by comparing multiple sequences that potentially contain the *cis*-regulatory binding sites sought after, based on the assumption that *cis*-regulatory binding sites are usually more conserved than their flanking non-functional sequences [3]. Therefore, the motif-finding problem is usually formulated to identify overrepresented segments of sequences from a set of input intergenic sequences that can be obtained by using a group of co-regulated genes in the same genome [4], or obtained by using a group of orthologous genes from multiple appropriately related genomes. The latter procedure is also called phylogenetic footprinting [5]. The motif-finding problem has promoted a large number of studies due to the importance of *cis*-regulatory binding sites in gene transcriptional regulation. Here we can only briefly review some of recently developed methods that are relevant to our method, for more intensive reviews, see [6-8].

Motif-finding algorithms can be largely categorized into "word enumeration" based and "pattern recognition" based methods. The former methods use different strategies to exhaustively enumerate *k*-mers in the input sequences. For example, MDScan [9] employs a deterministic greedy algorithm and Weeder [10] uses a suffix tree for the enumeration. Moreover, WINNOWER [11], CUBIC [12], cWINNOWER [13] and MotifCut [14] use graph-theoretic methods for the enumeration. Particularly, MotifCut represents each *k*-mer in the input sequences as a vertex and searches for maximum density subgraphs as motifs using a minimum-cut algorithm [14]. The more recently developed MoSDi algorithm [15] attempts to identify IUPAC motifs by exhaustively searching a fraction of the motif space according to a compound Poisson approximation of the distribution of the number of motif occurrences. The major drawback of word enumeration based methods is their computational inefficiency and application limitation to short motifs.

On the other hand, pattern recognition based methods often employ a probabilistic model for the representation of binding sites in the form of position-specific scoring matrices (PSSM) and an optimization procedure to find motifs. For example, Gibbs sampler [16], AlignACE [17], MotifSampler [18], and BioProspector [19] use a Gibbs sampling strategy to search the PSSM space, whereas MEME [20] uses an expectation-maximization (EM) strategy to find overrepresented sequences as possible binding sites. However, these optimization methods are known to be affected by local optima and can be very sensitive to small changes in the input sequences [6]. Recently, DEME [21], Seeder [22] and MoAn [23] were developed to find "discriminative" motifs, in which a motif is treated as a feature that leads to a good classification between a positive set of sequences containing binding sites and a negative (background) set of sequences [24]. However these algorithms are limited because they can only find motifs that exist in one group of sequences but not in another group of sequences. Furthermore, when the distribution of nucleotides in a motif is not significantly different from that of the background sequences, the probabilistic model based algorithms may fail to find the binding sites (see Results).

In addition, it has been shown that a consensus sequence and PSSM may not fully capture all the subtleties of a DNA binding motif of a TF, while more complex models that incorporate position-dependence of binding sites may over fit the input sequences [25]. Furthermore, the binding sites of a TF can be so degenerate that they can be divided into multiple distinct sub-motifs. For example, the experimentally verified 248 binding sites of the TF CRP in *E. coli *K12 can be divided into at least three sub-motifs; i.e., a more information content-rich canonical palindromic sub-motif, a T-rich sub-motif, and an A-rich sub-motif although both the latter sub-motifs share a certain number of elements with the canonical sub-motif [26]. Therefore, instead of predicting all the binding sites of a TF as a single motif, one should predict the binding sites of a TF as multiple sub-motifs and then merge them if possible. Since individual binding sites in such a sub-motif are likely to be highly similar to one another, if we treat each binding site in a sub-motif as a vertex of a graph, and connected two binding sites by an edge if their similarity is above a cut-off value, then these binding sites are likely to form a clique (complete subgraph). In other words, a sub-motif can be modelled as a clique from a graph-theoretic perspective. Therefore, finding sub-motifs of a TF is equivalent to finding some maximal cliques in a graph that represents the similarity of *k*-mers in a set of intergenic sequences. In this paper, we present a polynomial-time algorithm, MotifClick, for the problem based on this formulation while considering the distributions of nucleotides in binding sites and their background sequences as well as other statistical properties of binding sites (see Methods). We have tested MotifClick using both synthetic and real datasets of prokaryotes and eukaryotes, and found that our algorithm almost always outperforms existing leading algorithms in prediction accuracy on all datasets tested and is more computationally efficient than other graph-theoretic based algorithms such as MotifCut [14]. In addition, MotifClick seems to be the best among the leading algorithms at balancing the prediction sensitivity and specificity. More importantly, MotifClick is more likely than the existing tools to identify binding sites that have a similar nucleotide distribution to that of their background sequences.

### Overview of the MotifClick algorithm

**Figure 1:**
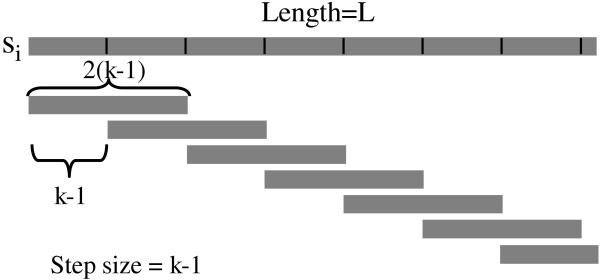
**Breakdown of an input sequence into a set of 2(*k*-1)-mers**. Each pair of adjacent 2(*k*-1)-mers overlap with each other by *k*-1 letters. Each 2(*k*-1)-mer will be used as a vertex to construct a graph.

Before giving a formal description of the MotifClick algorithm in Methods, we first outline its basic idea. To find a motif with length *k *in a set of sequences, MotifClick first converts each of the input sequences into a collection of 2(*k *- 1) -mers (Figure 1). Specifically, for each sequence, we extract all 2(*k *- 1)-mers with a step size *k *-1 (the last string may be shorter than 2(*k *- 1)), so that two adjacent 2(*k *- 1)-mers have an overlap of *k*-1 bases (Figure 1). We construct a graph *G *= (*V*, *E*), in which the vertices in *V *represent the 2(*k *- 1)-mers from all the input sequences. We connect each pair of vertices *u *and *v *by an edge with a weight *w*(*u*, *v*) if *w*(*u*, *v*) is above a cutoff *α*. The weight measures the maximum similarity of all local gapless alignments of *k*-mers between two 2(*k *- 1) -mers. Clearly, if the binding sites of a TF are highly conserved, then they can be represented by a clique or a high-density subgraph [11,14]. However, as we mentioned earlier, a TF can bind a few distinct motifs with little similarity. This fact promoted us to re-define the motif-finding problem as the search for multiple maximal weighted cliques in the graph *G *= (*V*, *E*) followed by merging them if possible.

**Figure 2:**
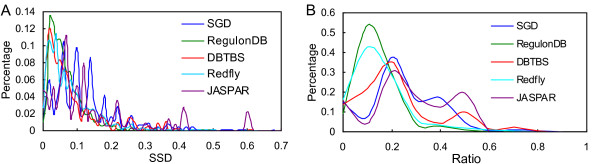
**Analysis of binding sites in five databases**. (A). Distributions of the sum of squared distance (SSD) between the nucleotide distribution of each motif and that of its background sequences in five databases, SGD, RegulonDB, DBTBS, Redfly, and JASPAR. (B). Distributions of the ratio of maximum length of a segment of single-nucleotide in a binding site to the length of the binding site in the five databases.

Furthermore, to measure the significance of difference between a binding site and its background sequence, we define the *sum of squared distance *(SSD) between the nucleotide distribution {*p*(*b*)} of each binding site and that of its background intergenic sequence {*q*(*b*)} as . Through analyzing the motifs collected in five databases, SGD [27], RegulonDB [28], DBTBS [29], Redfly [30] and JASPAR [31], we found that a considerable portion of binding sites in the five databases have an SSD < 0.2 (Figure 2A). Therefore, some binding sites in both bacteria and eukaryotes may not have a significantly different distribution of nucleotides frequency from that of their background intergenic sequences. This might explain why it is often difficult to find motifs whose elements have a small SSD using existing motif finding tools [32] (see Results and discussion). To overcome this shortcoming of existing tools, we take SSD into account in our algorithm.

## Results and discussion

To evaluate the performance of MotifClick, we first compared it with four leading general purpose motif finding tools: BioProspector [19], MEME [20], MotifCut [14], and Weeder [10] on both synthetic and real datasets. We selected these tools for the comparison based on recent survey studies [32,33] and our own evaluation of multiple motif-find tools [26]. For instance, Tompa *et al*. [32] assessed 13 motif-finding tools for the discovery of binding sites in eukaryotes using a dataset from the TRANSFAC database, and found that Weeder outperformed the others for most measures, and that MEME also performed well although not as good as Weeder. In another survey study, Hu *et al*. [33] evaluated five tools, AlignACE [17], MEME, BioProspector, MDScan [9], and MotifSampler [18], for the prediction of binding sites in prokaryotes using a dataset from RegulonDB [28], and found that MEME often achieved the highest sensitivity, while BioProspector often had the highest specificity. We included MotifCut for the comparison in the current study because it is also a graph-theoretic algorithm and is claimed to discover more known yeast motifs than do MEME and BioProspector. We also evaluated an option (-s 0) of our MotifClick program for finding more degenerate binding sites than the default setting. We excluded a few new methods, Seeder [22] and DEME [21], because they do not support the mode 'any-number of repetitions' (anr); MoAn [23], because it requires a large and reasonable negative set; and MoSDi [15], because its output format is IUPAC, making the comparison difficult.

Furthermore, with the availability of increasing numbers of sequenced genomes, phylogenetic footprinting has proved to be a powerful method for predicting *cis*-regulatory binding sites in both eukaryotic [34] and prokaryotic genomes [5]. In this approach, one identifies well-conserved segments of sequences as potential binding sites from the intergenic sequences of a set of orthologous genes from a group of appropriately related genomes based on the assumption that binding sites of orthologous genes are largely conserved in closely related genomes. Several existing general purpose motif-finding algorithms such as AlignACE, Gibbs sampler, MEME, and CONSENSUS [35] have been used for phylogenetic footprinting in both eukaryotes and prokaryotes [36-40]. We [26] have recently evaluated six tools, BioProspector, CONSENSUS, CUBIC [12], MDScan, MEME and MotifSampler for the prediction of binding sites in prokaryotes by phylogenetic footprinting, and found that MEME and BioProspector recovered more known binding sites than the others. Therefore, we also compared the MotifClick algorithm with the four leading general purpose motif-finding tools for phylogenetic footprinting on both prokaryotic and eukaryotic datasets.

In addition, motif-finding algorithms that are specifically for phylogenetic footprinting have also been developed, such as FootPrinter [41,42], WeederH (Weeder for homologous sequences) [43], phylogenetic Gibbs sampler [44], PhyloGibbs [45], PhyME [46], and CompareProspector [47]. These algorithms typically take into account the phylogenetic relationships of the input sequences when identifying the most conserved *k*-mers. Thus, besides the four leading general purpose motif-finding tools mentioned above, we also compared our algorithm with FootPrinter and WeederH. However, we excluded other phylogenetic motif-finding tools such as phylogenetic Gibbs sampler, PhyloGibbs, PhyME, and CompareProspector, because their input orthologous intergenic sequences need to be aligned by a multiple alignment tool, but many orthologous intergenic sequences are not alignable. We also excluded phylogenetic motif-finding tools such as PhyloCon [48] and PRIORITY [49] because they were designed to search for motifs that are both overrepresented in a set of sequences from a genome and conserved across related organisms (the so-called 'multiple genes, multiple species' approach).

### Evaluation on synthetic datasets

In order to obtain accurate false positive rates and avoid possible overtraining of the algorithm parameters, we evaluate MotifClick along with the four selected general purpose tools using the synthetic datasets.

#### 1. Motifs with a length of eight bases

**Figure 3:**
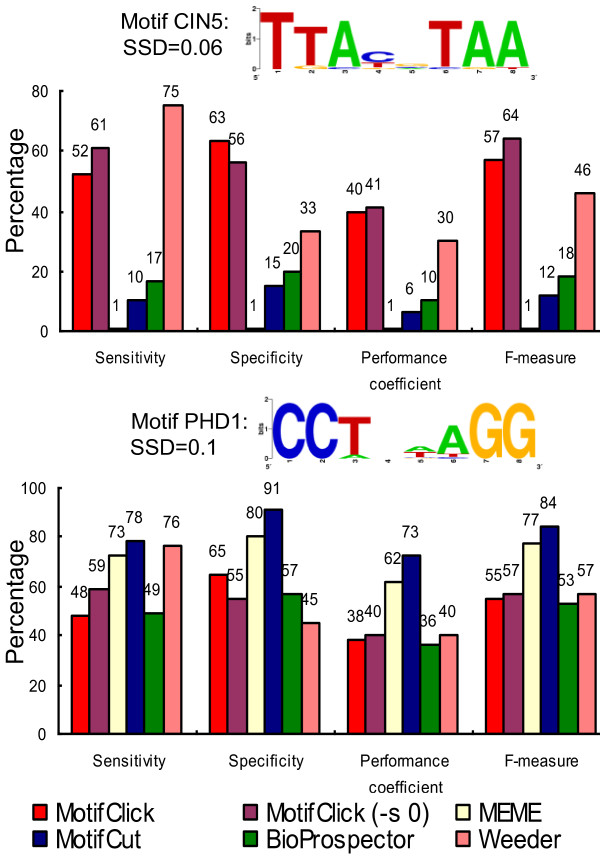
**Comparison of MotifClick with the default setting and the option (-s, 0) with four other tools for predicting binding sites of two 8-mer yeast motifs, CIN5 and PHD1**. The binding sites of both motifs were separately implanted in two sets of 20 400-base synthetic sequences.

We first evaluated our algorithm for identifying motifs with a length of eight bases implanted in the synthetic background sequences. In order to assess the ability of the tools for finding motifs with different SSDs, we selected two motifs from the SGD database [27], CIN5 and PHD1, each contains 20 binding sites, with an average SSD of 0.06 and 0.1 respectively. We selected these two motifs for the evaluation since the majority of yeast binding sites have an SSD = 0.06 or SSD = 0.1 (Figure 2A). Binding sites are implanted in 400-base background sequences. As shown in Figure 3, for the motif CIN5, Weeder has the highest sensitivity, while MotifClick achieves the highest specificity, performance coefficient, and F-measure. However, for the motif PHD1, MotifCut outperforms the four other methods. Notably, MotifClick is the best at balancing specificity and sensitivity for both motifs. Furthermore, MotifClick and Weeder have a more consistent performance than the other methods on both motifs. This result might suggest that MotifClick performs better when the SSD is small. To further confirm this speculation, we evaluated MotifClick with the SSD option set to 0.06 and the other tools on more 8-mer motifs with an SSD ≤ 0.06 (e.g., CIN5, FKH1 and GCN4 from the SGD database) that are implanted in synthetic background sequences. As shown Table S1 in Additional file 1, MotifClick generally has the smallest log-odds ratios with respect to the other programs, suggesting that it is able to find the most binding sites that are missed by the other tools.

**Figure 4:**
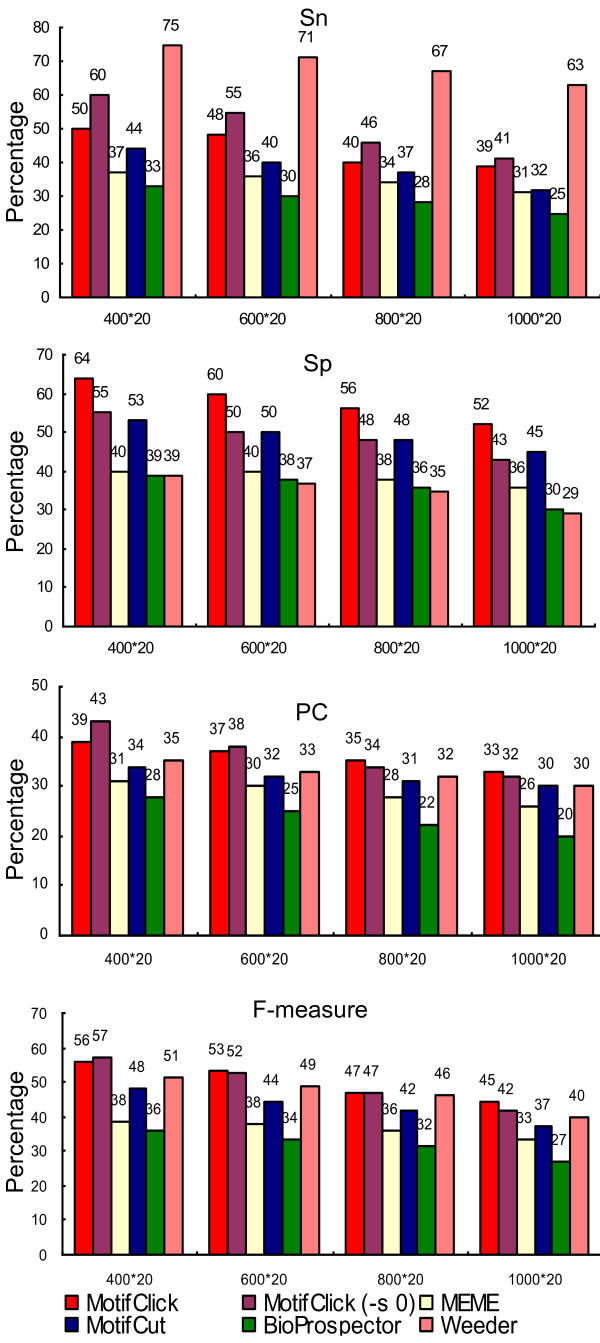
**Comparison of MotifClick with four other algorithms for predicting binding sites from yeast and fly implanted in synthetic sequences**. In these plots the X-axis represents the input size (the number of nucleotides in a sequence times the number of sequences) and the Y-axis the percentage of correctly identified binding sites.

To further compare MotifClick with other tools for their ability to find binding sites located in different sizes of background sequences, we selected six motifs with a length of eight bases from SGD and six motifs with a length of eight bases from Redfly for the evaluation. All these motifs have an SSD < 0.15, and are available at http://motifclick.uncc.edu. Each binding site was implanted in a background sequence of a length of 400, 600, 800 and 1,000 bases. The average performance of each tool on the 12 motifs in each background sequence size (400 × 20, 600 × 20, 800 × 20 and 1,000 × 20) is shown in Figure 4. Again, MotifClick achieves the highest specificity, performance coefficient, and F-measure while Weeder has the highest sensitivity, but the lowest specificity on each dataset among the programs. It should be pointed out that the default parameters were used for all the programs; however, we noted that the default of Weeder could search eight-base motifs with two mutations while the other programs did not specify the number of mutations allowed, so it may be unfair to the other programs.

#### 2. Motifs with a length of 16 bases

**Figure 5:**
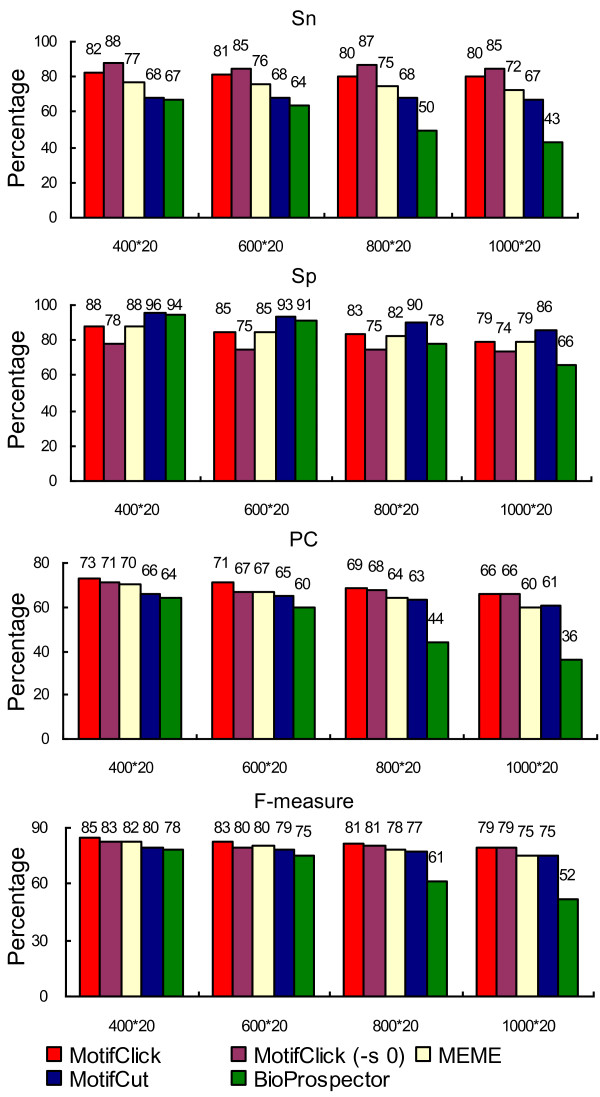
**Comparison of MotifClick with other three algorithms for predicting binding sites from *E. coli *K12 and *B. subtilis *implanted in synthetic sequences**. In these plots, the X-axis represents the input size (the number nucleotides in a sequence times the number sequences) and the Y-axis the percentage of correctly identified binding sites.

We then evaluated our algorithm for identifying binding sites with a length of 16 bases taken from RegulonDB and DBTBS and implanted in background sequences of different sizes. Now that the vast majority of motifs in RegulonDB and DBTBS have an SSD < 0.1 (Figure 2A), we chose 10 motifs from RegulonDB and eight motifs from DBTBS with an average SSD < 0.1. These motifs and background sequences are available at http://motifclick.uncc.edu. Weeder was not evaluated on these datasets, because it only accepts motif lengths of even values between 6 and 12. As shown in Figure 5, MotifClick achieves the highest sensitivity, performance coefficient and F-measure, while MotifCut has the highest specificity for all the datasets of different background sequence sizes.

To evaluate the noise tolerance of our algorithm, we added 5 and 10 additional background sequences (noises) of size 400 bases to each of the sequence sets in the 400 × 20 datasets constructed above for the 18 motifs, to form two datasets denoted as 400 × 25 (25% added noises) and 400 × 30 (50% added noises), respectively. No binding sites were implanted in any of these 5 or 10 additional sequences; therefore, they increased the noise levels in the original sequence sets. As shown in Figure S1 in Additional file 1, with increasing levels of noise, MotifClick almost constantly outperforms the other programs, and the noise levels have only a small effect on its performance for all the evaluations. Therefore, besides its outstanding performance for prediction accuracy, our program is also rather noise-tolerant, comparable to the best tools evaluated such as MEME.

Furthermore, we found that all the motif-finding tools can accurately indentify motifs with an SSD > 0.1, such as CRP, LexA, and PhoB in RegulonDB, because they are easy to be separated from background. However, when evaluating MotifClick with the SSD option set to 0.06 and the other tools on the synthetic datasets implanted with the 16-base motifs having an SSD ≤ 0.06, such as ArgR, CpxR, H-NS, GerE, and PhoP, we found that MotifClick outperforms the other tools for identifying these motifs with an SSD ≤ 0.06, and can predict the most binding sites that are missed by other tools as indicated by its smallest log-odds ratios with respect to the other tools (Table S2 in Additional file 1).

We evaluated the running time of MotifClick on an Intel Xeon processor using all the synthetic datasets described earlier with motifs of lengths 8, 12, and 16 implanted in sequence sets with nucleotide sizes 400 × 20, 600 × 20, 800 × 20, and 1000 × 20. As shown in Figure S2 in Additional file 1, the running time of the program is from several seconds to a few minutes on these datasets, and increases almost linearly with the size of the input sequences. Thus, MotifClick's computational efficiency is also comparable to that of the other algorithms; especially it runs much faster than MotifCut though both are based on graph theory. For instance, it only takes MotifClick a few minutes (on average, about 5.7 minutes) to find a 16-mer motif in each dataset consisting of 1000 × 20 nucleotides (Figure S2 in Additional file 1), while MotifCut needs more than 30 minutes, on the Intel Xeon processor. The main reason is that MotifClick only searches a portion of 2(*k*-1)-mers while MotifCut uses all possible *k*-mers as vertices.

### Evaluation on real datasets

#### 1. Sequences from the same genome

**Figure 6:**
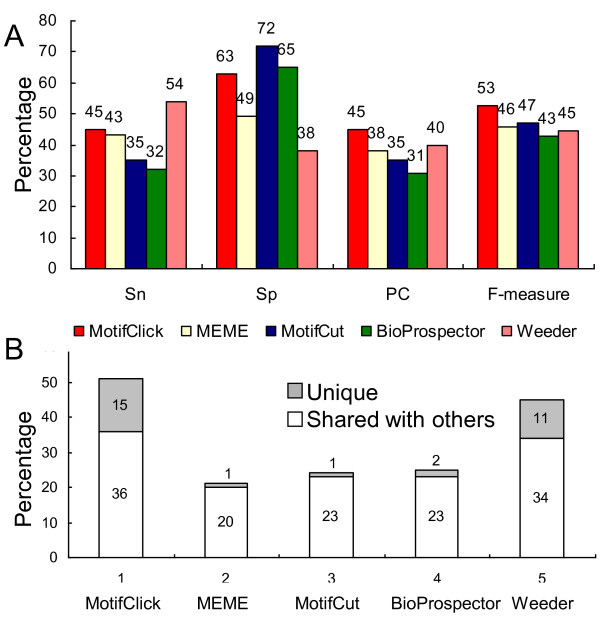
**Comparison of MotifClick with four other algorithms on real datasets**. (A) The results are the average performance of each algorithm on the 60 selected motifs located in their native genomic sequences. (B) The percentage of binding sites uniquely recovered by each algorithm and the percentage of binding sites shared with the four other algorithms on the real dataset of 20 motifs with an SSD ≤ 0.06.

To evaluate our algorithm on real datasets, in addition to the 30 motifs that we have selected (10 from RegulonDB, eight from DBTBS, six from SGD, and six from Redfly), we selected another 30 motifs with lengths of 8~16 bases from JASPAR [31], each contains at least five binding sites. These 60 motifs were placed back in their original locations of genomic sequences of a maximum length of 1,000 bases. To make the datasets more coherent, we removed binding sites that contain more than 30% degenerate bases and have an SSD > 0.3. However, we kept all binding sites in each sequence if there are two or more binding sites in it. These motifs and genomic sequences are available at http://motifclick.uncc.edu. As shown in Figure 6A, MotifClick achieves the highest PC and F-measure, while Weeder has the highest sensitivity, and MotifCut has the highest specificity. Therefore MotifClick is also the best at balancing sensitivity and specificity on the real datasets.

In order to evaluate the sensitivity of our program to the values of the option '-SSD' specified by a user, we ran the program on the 60 real datasets using eight different '-SSD' values in the range of [0.15, 0.5]. As shown in Figure S3 in Additional file 1, although the binding sites of the 60 motifs have an SSD in the ranger of [0.05, 0.3], the performance of MotifClick only slightly decreased when the value of the option '-SSD' deviated from that of the motifs in the dataset, in particular when the former is larger than the latter. However, one should avoid using a smaller value of '-SSD' when the motif to be found may have a higher SSD. To further evaluate the ability of MotifClick to identify motifs with a low SSD that may be missed by other algorithm, we ran MotifClick with the SSD option set to 0.06 and the four other algorithms on 20 motifs with an SSD ≤ 0.06 from the 60 motifs of real datasets. As shown in Table 1, MotifClick generally had the smallest log-odds ratio with respect to each of the other algorithms. Therefore, MotifClick is indeed the best at predicting motifs with a low SSD that are missed by other algorithms (Figure 6B).

**Table 1:** Log-odds ratios of pairs of programs for predicting binding sites with an SSD ≤ 0.06 in the real datasets

	BioProspector	MEME	MotifCut	Weeder
MotifClick	0.21	0.18	0.23	0.27
Weeder	0.28	0.31	0.29	
MotifCut	0.37	0.26		
MEME	0.46			

#### 2. Phylogenetic footprinting datasets from fungi and bacteria

To benchmark the performance of our algorithm for phylogenetic footprinting on real data, we compared MotifClick with the four general purpose motif-finding tools as well as two phylogenetic motif-finding tools, WeederH and FootPrinter, on orthologous intergenic sequence sets from the yeast (*S. cerevisiae*) genome and other six closely related fungal genomes, as well as orthologous inter-operonic sequence sets from the *E. coli *K12 genome and other 55 closely related γ -proteobacterial genomes.

##### 2.1 Yeast data

Because almost all of the seven motif-finding tools require to specify a length of the motifs to be predicted, and the majority of known binding sites in yeast have a length of about eight bases, we chose eight bases as the fixed motif length for motif-finding for all the tools except for Weeder and WeederH, for the former we used the option 'medium' as the motif length, and for the latter we used the default options. We applied each program in the 'anr' mode if available to the 5,137 orthologous intergenic sequence sets from the fungal genomes (see Methods), and considered the best predicted (top1), top 5, top 10, top 15, and top 20 motifs for the evaluation. We used the predictions from the yeast genome for the evaluation, because there are 2,932 known binding sites belonging to 99 TFs from the genome in the input intergenic sequences sets. However, since not all binding sites in the intergenic sequences in yeast are known, to evaluate prediction specificity, we define the low bound of specificity (lbSp) as the number of recovered known binding sites divided by the number of predicted sites. As shown in Table 2, Weeder recovers the highest number of known binding sites, but achieves the lowest lbSp, and BioProspector achieves the highest lbSp but recovers the fewest number of known binding sites among these programs for most of the top numbers of predictions. On the other hand, the results in Table 2 clearly show that MotifClick outperforms MEME and MotifCut in terms of both the coverage of known binding sites and lbSp. Furthermore, although Weeder recovers more known binding sites than does MotifClick for each top number of predictions, this is at the cost of its low prediction specificity. For instance, the number of predicted sites (24,916) in the best motifs (top 1) returned by Weeder is very close to that (23,417) of the top 5 motifs returned by MotifClick, but MotifClick recovers more known binding sites in its all top 5 motifs than does Weeder in its all best motifs (1,200 vs. 969). Therefore, Weeder is likely to have much more false positive predictions than the other tools, and MotifClick is the best at balancing the true positives and false positives. As shown in Table 2, the two phylogenetic motif-finding tools WeederH and FootPrinter do not perform very well on our dataset. The possible reason for WeederH could be that it only has the 'oops' mode ('one occurrence per sequence') while some of our input sequences contain more than one binding site.

**Table 2:** Evaluation on the phylogenetic footprinting datasets from fungal genomes using the predictions in the yeast genome

		Top 1	Top 5	Top 10	Top 15	Top 20
MotifClick	BS (MF)	585 (67)	**1200 **(85)	1638 (92)	1923 (95)	2107 (95)
	
	PS (lbSp)	7158 (**0.081**)	**24916 **(0.048)	41752 (0.039)	55084 (0.035)	65852 (0.032)

MEME	BS (MF)	754 (70)	**1202 **(85)	1615 (87)	1931 (92)	2087 (95)
	
	PS (lbSp)	10107 (0.074)	34010 (0.035)	49958 (0.032)	60805 (0.031)	69709 (0.030)

MotifCut	BS (MF)	474 (65)	1189 (85)	1641 (86)	1893 (93)	1983 (95)
	
	PS (lbSp)	7632 (0.062)	28074 (0.041)	47583 (0.034)	61107 (0.031)	67017 (0.030)

BioProspecter	BS (MF)	780 (79)	1145 (84)	1465 (86)	1701 (89)	1911 (92)
	
	PS (lbSp)	10049 (0.078)	20418 (**0.056**)	31935 (0.046)	42305 (0.040)	52296 (0.037)

Weeder	BS (MF)	**969 **(77)	**1698 **(88)	2063 (92)	2255 (94)	2396 (94)
	
	PS (lbSp)	**23417 **(0.041)	56440 (0.030)	81374 (0.025)	96046 (0.023)	106872 (0.022)

WeederH	BS (MF)	404 (64)	1154 (79)	1614 (89)	1905 (91)	2129 (94)
	
	PS (lbSp)	5092 (0.079)	25363 (0.045)	49929 (0.032)	74180 (0.026)	97300 (0.022)

FootPrinter	BS (MF)	452 (66)	1148 (78)	1579 (86)	1854 (91)	2073 (94)
	
	PS (lbSp)	6173 (0.073)	27451 (0.042)	48533 (0.033)	77249 (0.024)	86437 (0.024)

BS, number of recovered known binding sites; MF, number of recovered motifs; PS, number of predicted sites; lbSp = BS/PS, lower bound of specificity.

##### 2.2 Bacterial datasets

To compare our program with the other programs for phylogenetic footprinting using the orthologous interoperonic sequence sets from *E. coli *K12 and other 55 γ-protoebacterial genomes, we chose 16 bases as the fixed motif length for the motif-finding tools as most bacterial binding sites have a length of 12-22 bases [26]. Weeder, WeederH, and FootPrinter were not evaluated here as they are not designed to find a binding site longer than 12 bases. As shown in Table 3, MotifClick and BioProspector achieve the highest lbSp in most cases among these tools, but MotifClick recovers more known binding sites than does BioProspector. Moreover, MotifClick and MEME recover the largest number of known binding sites in most cases among these tools, but MotifClick has higher lbSp than does MEME. Therefore MotifClick is the best not only for binding site coverage but also for lbSp.

**Table 3:** Evaluation on the phylogenetic footprinting datasets from γ-proteobacterial genomes using the predictions in the *E. coli *K12 genome

		Top 1	Top 5	Top 10	Top 15	Top 20
MotifClick	BS (MF)	331 (85)	793 (108)	1055 (114)	1186 (114)	1262 (117)
	
	PS (lbSp)	2575 (**0.129**)	7706 (**0.103**)	11056 (0.095)	12779 (0.093)	13592 (**0.093**)

MEME	BS (MF)	298 (83)	877 (109)	1134 (115)	1202 (117)	1233 (117)
	
	PS (lbSp)	3352 (0.089)	14243 (0.062)	20999 (0.054)	23912 (0.050)	25412 (0.049)

MotifCut	BS (MF)	241 (75)	487 (89)	544 (96)	640 (102)	744 (107)
	
	PS (lbSp)	1942 (0.124)	4763 (0.102)	6552 (0.083)	9145 (0.070)	10408 (0.071)

BioProspecter	BS (MF)	354 (85)	743 (102)	953 (112)	1056 (112)	1150 (116)
	
	PS (lbSp)	4950 (0.072)	7678 (0.097)	10090 (**0.107**)	11287 (**0.094**)	12306 (**0.093**)

MotifClick + MEME	BS (MF)	474 (98)	1029 (114)	1259 (118)	1335 (120)	1357 (120)

MEME + BioProspector	BS (MF)	472 (92)	1051 (115)	1258 (118)	1312 (119)	1339 (119)

BS, number of recovered known binding sites; MF, number of recovered motifs; PS, number of predicted sites; lbSp = BS/PS, lower bound of specificity.

In addition, we also evaluated the complementary effects of MotifClick with other two best performers MEME and BioProspector. As shown in the last four rows of Table 3, the combination of MotifClick and MEME recovers more binding sites and motifs than the combination of MEME and BioProspector, suggesting again that MotifClick is well complementary with other tools.

## Conclusions

We have developed a novel graph-theoretic algorithm, MotifClick, for *cis*-regulatory binding site prediction, and have demonstrated that it outperforms several current leading motif-finding tools on both synthetic and real datasets, especially when the nucleotide distribution of a motif is similar to that of its background sequence. Furthermore, MotifClick is the best at balancing the prediction specificity and sensitivity among the tools evaluated. In addition, MotifClick is well complementary with a few other leading motif-finding tools, thus can be used in combination with these tools to improve binding site predictions in ensemble algorithms such as WebMOTIFS [50], EMD [51], GLECLUBS [26] and eGLECLUBS[52].

In the perspective of algorithm design, MotifClick has several main advantages over most existing methods in general: (a) Besides largely reducing the graph size, our use of 2(*k *- 1) -mers avoids the drawbacks of directly using *k*-mers by other algorithms, caused by the fact that two binding sites of a TF may sometimes have fewer matches than one of their neighboring *k*-mers. (b) The merged cliques generated by our algorithm can capture more binding sites of a TF than the consensus and PSSM models, in particular, when the binding sites of a TF can be divided into distinct sub-motifs. (c) Although each clique is found by a greedy strategy, the enumeration of maximal cliques can guarantee our algorithm not to be trapped in a local optimum. And (d) since we ignore some *k*-mers that are less likely to be true binding sites, although MotifClick is a word-based method, it does not exhaustively enumerate all the *k*-mers.

## Methods

### Datasets

#### 1. Binding sites and their background sequences

Binding sites from five databases SGD [27], RegulonDB [28], DBTBS [29], Redfly [30], and JASPAR [31] were downloaded from their respective websites. The background sequences (i.e., genomic sequences in which the binding sites are located) of binding sites were downloaded from the NCBI ftp server (ftp://ftp.ncbi.nih.gov).

#### 2. Synthetic datasets

Tompa *et al*. [32] have designed three benchmarks, i.e., the 'real', the 'generic', and the 'Markov' benchmarks using 56 eukaryotic motifs from TRANSFAC for evaluating the performance of motif-finding tools. More recently, Sandve *et al*. [53] designed an updated suite of three improved benchmarks using 114 motifs from TRANSFAC. However, it is probably inappropriate for us to employ Tompa's or Sandve's benchmarks to compare MotifClick with other tools because both benchmarks were created using only eukaryotic binding sites, and each sequence in Sandve's benchmarks contains exactly one binding site and MotifClick is not designed for the mode 'one occurrence per sequence' (i.e., 'oops'). Therefore, in order to test our algorithms in the 'any-number of repetitions' ('anr') mode and on finding binding sites of both prokaryotes and eukaryotes, we have designed our own synthetic datasets and real datasets while only using Sandve's benchmarks to train some cutoffs in the MotifClick algorithm.

To construct synthetic datasets for the evaluation of our algorithm, we selected motifs with a length of eight and 16 bases and information contents about 12 (i.e., almost six positions are conserved) and 16 bits (i.e., almost eight positions are conserved), respectively, from the downloaded datasets. Each motif consists of 20 binding sites. If the original motif contains more than 20 binding sites, then the binding sites were selected to maintain the level of information contents mentioned above. These two motif lengths were chosen for the evaluation, because the majority of eukaryotic motifs have a length from seven to 10 bases, and the majority of prokaryotic motifs have a length from 12 to 22 bases. In an experiment, the 20 binding sites in a motif are implanted in 20 background sequences of length 400, 600, 800 and 1,000 bases, respectively, by randomly replacing a segment of original sequence of the same size of the binding site. Specifically, for each background size, each of the 20 binding sites of a motif was randomly implanted in one of 20 background sequences generated by a third-order Markov model; some sequences may be implanted with more than one binding site (i.e., the 'anr' mode), while some sequence may not be implanted with any binding site at all. The transition probabilities of the third-order Markov models were estimated from all intergenic sequences of the host genomes (*Saccharomyces cerevisiae *for the database SGD, *Drosophila melanogaster *for Redfly, *E. coli *K12 for RegulonDB, and *B. subtilis *for DBTBS). For each background size, the results are the statistics of 100 repeated experiments of each motif with each binding site in the motif being implanted in 100 different sets of background sequences.

#### 3. Phylogenetic footprinting datasets

##### 3.1 Fungal datasets

The intergenic sequence files of yeast (*S. cerevisiae*) and other six fungal genomes (*S. bayanus*, *S. castellii*, *S. kluyveri*, *S. kudriavzevii*, *S. mikatae*, and *S. paradoxus*) were downloaded from the SGD ftp server (ftp://genome-ftp.stanford.edu/pub/yeast/data_download/sequence/fungal_genomes). The orthologous groups in SGD were compiled from two previous studies [34,54]. For each group of orthologous genes, we extracted up to 1,000 bases upstream intergenic region of each gene to form an orthologous sequence set. After deleting the sequence sets that contain fewer than five orthologs, we obtained 5,137 orthologous sequence sets, which contain 2,932 known binding sites belonging to 99 TFs in *S. cerevisiae*.

##### 3.2 Bacterial datasets

A total of 2,313 orthologous inter-operonic sequence sets from *E. coli *K12 and other 55 closely-related γ-proteobacterial genomes were taken from our previous work [26]. Orthologous genes between two genomes were predicted by bidirectional best hits (BDBH) method [55] using BLASTP with an E-value cutoff 10^-20 ^for both searches. These sequences contain 1,411 known binding sites belonging to 122 TFs in *E. coli *K12 according to the RegulonDB v6.0 database [28]. All the datasets are available at: http://motifclick.uncc.edu.

### The algorithm

### 1 Graph construction

Let *S*_1_, *S*_2_,... *S*_*n *_be the set of input sequences, and each sequence *S*_*i *_be a string of nucleotides (*a*_*i*1_,..., *a*_*iL*_). For each *S*_*i*_, we break it into a set of 2(*k *- 1)-mers {*s*_*ij *_= (*a*_*i*,(*k*-1)*j*+1_,...,*a*_*i*,(*k*-1)(*j*+2)_)} for all possible *j *(*j *= 0, 1, ... ; the last string may be shorter than 2(*k *- 1), but must be longer than *k *- 1) (Figure 1). Because any two such adjacent strings have (*k *- 1) overlapping positions, any *k*-mer in *S*_*i *_can be located in only one of the 2(*k *- 1)-mers. We construct a graph *G*=(*V*,*E*) using all of the strings {*s*_*ij*_} as the set of vertices *V=*{ *v*_*ij *_}. We connect any two vertices *v*_*ij *_and *v*_*ht *_if the weight of the edge is greater than a cutoff *α*. Therefore, *G *is a graph in which any two vertices from any sequences (even the same sequence) can be joined by an edge. We now describe how to assign an appropriate weight to each edge.

Given two vertices *v*_*ij *_and *v*_*ht*_, let *K*_*ij *_and *K*_*ht *_be the sets of all *k*-mers in the corresponding 2(*k *- 1)-mers *s*_*ij *_and *s*_*ht *_respectively. The weight *w*_*ijht *_between *v*_*ij *_and *v*_*ht *_is defined as:(1)

where *M*(*a*, *b*) is the number of matches between a pair of *k*-mers *a *and *b*. That is, the weight of an edge in *E *is defined as the maximum number of matches of all of *k*-mers in the corresponding two 2(*k *- 1)-mers. However, we exclude some *k*-mers in the strings *s*_*ij *_and *s*_*ht *_from the sets *K*_*ij *_and *K*_*ht*_, respectively, based on the following observations. First, as shown in Figure 2A, over 95% known binding sites in the databases SGD [27], RegulonDB [28], DBTBS [29], Redfly [30] and JASPAR [31] have an SSD < 0.3. Second, as shown in Figure 2B, more than 97% binding sites in the five databases have the longest single-nucleotide segments shorter than 60% of the binding sites. Third, we also observe that more than 95% binding sites in the five databases consist of at least 3 of the 4 types of nucleotides A, C, G and T (data not shown). Therefore, we exclude in *K*_*ij *_the *k*-mers 1) that have an SSD > 0.3, 2) that have a single-nucleotide segment longer than 60% of its length, or 3) that consist of at most 2 types of nucleotides. In the MotifClick program, we provide an option '-SSD' that allows a user to adjust the cutoff of SSD (the default cutoff is 0.3) in order to find motifs that have a similar nucleotide distribution to that of their background sequences.

In order to choose a proper weight cutoff *α *for creating the edge set *E*, we first randomly select a number *N *= max{10, *n*/4}of *k*-mers from the sequence set *S*_1_, *S*_2_, ..., *S*_*n*_. For each of such *k*-mer *a *and each input sequence *S*_*i*_, we calculate the maximum number of matches *M*(*a*, *S*_*i*_) between *a *and *S*_*i *_by scanning *S*_*i *_with *a *(if *a *is from *S*_*i*_, we skip the location of *a *in *S*_*i*_.). Therefore, we have (*n*-1)*N *of such values. We calculate the average value  of these match numbers after removing 5% minimum ones. To test possible bias of our sampling process for computing , we ran the sampling procedure 1000 times on a set of 1000 × 40 input sequences to find 8-mers, 12-mers, and 16-mers, respectively, and found that  values are identical for more than 99% of samples (data not shown). Therefore, our sampling strategy is not biased and sufficient. We assume that the average number of matches among the binding sites of a TF should be larger than that of the randomly selected *k*-mers. Then we set the default weight cutoff . In the MotifClick program, we also add an option (-s 0) for predicting more degenerate binding sites by setting .

#### 2 Finding and merging cliques

**Figure 7:**
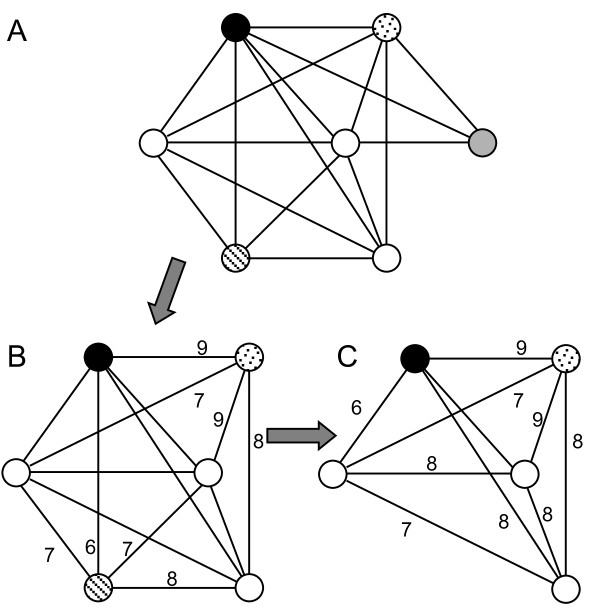
**The procedure for finding the maximal clique associated with the black vertex in its neighborhood subgraph**. We first delete the grey vertex that has the minimum degree, and then break the tie of the dotted and streaked vertices by deleting the streaked one as it has a smaller sum of weights on the incident edges.

A maximal clique is a clique that is not included in a larger clique in a graph. Listing all of the maximal cliques (the total number is an exponential function of the number of vertices) and finding the maximum clique in a graph is NP-hard [56]. Therefore, instead of finding the maximum clique, we intend to find a maximum clique associated with each vertex *v *in the graph *G *= (*V*,*E*). To this end, we define the neighborhood subgraph *N*(*v*) of a vertex *v *as the subgraph induced by *v *and its neighbor vertices. Clearly, all maximal and maximum cliques containing the vertex *v *must be in *N*(*v*). We use a greedy strategy to find exactly one maximal weighted clique associated with *v *in *N*(*v*) by sequentially deleting its minimum-degree neighbor vertex until the degrees of the remaining vertices are equal to one another. If at least two vertices have the same minimum degree, we break the tie by deleting the one with the minimum sum of weights of its incident edges. An example of using this algorithm to find a clique is shown in Figure 7. Note that this greedy strategy cannot guarantee the resulting clique is the maximum clique of the neighborhood subgraph *N*(*v*), but it can guarantee that the clique is a maximal one. The time complexity of the procedure is *O*(*d*^2^(*v*)) if the degree of the vertex *v *is *d*(*v*). Since the graph *G *is usually sparse, this greedy strategy is rather efficient. In practice, to ensure the sparsity of the graph, we reset *α *= α + 1 until *D *is smaller than the option value if the graph density *D *= | *V* | / | *E* | is too high.

Clearly, by applying the greedy algorithm to the neighborhood graphs of all vertices, we can obtain |*V*| maximal cliques. Therefore, this greedy algorithm ensures that each vertex is included in at least one of the resulting cliques, and it cannot be trapped into a local optimum. However, there may be overlapping or even redundant cliques in the |*V*| maximal cliques. Moreover, as we indicated earlier, cliques are too strict for clustering the binding sites of the same TF, as the binding sites of the same TF can be divided into different cliques due to the low similarity of different sub-motifs of the TF. Therefore, we need to combine these cliques if possible.

To this end, we first select the clique with the maximum sum of edge weights as a core clique, denoted by *C*. We initialize the group of merged cliques *MC*: = {*C*}. For each of the other cliques *C'*, we calculate two overlapping rates between C and *C'* as follows:(2)

If either r & ≥ γ or R & ≥ *β*, where *γ *and *β *are two preset cutoff values, and *γ *>*β *because in most cases | *C'* | ≤ | *C *|, then we set *MC*: = *MC *∪ {*C'*}. We obtain the final group of cliques *MC *by traversing all cliques. We tested our program with different combinations of *γ *and *β*, where *γ *and *β *took values of 1/2, 2/3, 3/4, 3/5, 4/5, and 5/6, on the three benchmark suites ('algorithm Markov', 'algorithm real', and 'model real') proposed by Sandve *et al*. [53]. We found that the algorithm achieved the highest average nucleotide-level correlation coefficient (nCC) when (*γ*,*β*) = (3/4, 3/5) for the three suites (the average nCC = 0.062). The two cutoffs can be understood intuitionally: if *C*' is a 4-vertex clique sharing a triangle (3-vertex clique) with *C*, or *C *is a 5-vertex clique sharing a triangle with *C'*, then *C *and *C*' can be merged together.

#### 3 Aligning and refining sequences in merged cliques

The sequences in merged cliques *MC *are 2(*k *-1)-mers, but some of them may not contain real binding sites. To find a true *k*-mer motif in *MC*, we need to filter out the spurious ones. To this end, we construct a multipartite graph *C*' by connecting any two *k*-mers from two different 2(*k *- 1)-mers in *MC *if the number of matches between them is not less than . We identify the largest neighborhood graph N* as the final motif by enumerating the neighborhood subgraph *N*(*v'*) of each vertex *v*' in *G'*. If at least two neighborhood subgraphs have the same largest size, we break the tie by selecting the one with the maximum sum of edge weights. The similarity between each pair of *k*-mers in N* is generally high because of the high density of the merged cliques *MC*.

In practice, one often wants to find more than one motif from a set of input sequences. We achieve this goal by repeatedly applying the merging and refining steps described above on the remaining maximal cliques after removing those that are merged into *MC*. Notably, a sequence could appear in different top motifs due to its appearance in multiple cliques. However, any two returned motifs usually cannot overlap with each other in a large scale because of the clique-merging step.

### Performance and complementarity measurements

We consider that a binding site is identified by a motif-finding tool if at least 50% nucleotides of the binding site overlap with the predicted sequence. For the evaluations on the synthetic datasets, we define the following accuracy metrics: (1) Sensitivity: Sn = TP/(TP + FN), (2) Specificity: Sp = TP/(TP + FP), (3) Performance coefficient: PC = TP/(TP + FP + FN), and (4) F-measure or Harmonic mean: F = 2*Sn*Sp/(Sn + Sp), where TP, FP, and FN are the numbers of true positive, false positive, and false negative predictions, respectively. PC is an integrated measurement of sensitivity and specificity, while F-measure is an overall performance measurement. Hu *et al*. [33] have shown that PC has several advantages over correlation coefficient (CC), and that F-measure tends to penalize more the imbalance of sensitivity and specificity than does geometric or arithmetic mean.

We hope that MotifClick can identify some binding sites that are missed by other algorithms, in particular, when the SSD of a binding site is low, thus it is complementary to these tools. To evaluate the complementarity of the predictions of two tools, we compute a log-odds ratio as used in MotifCut [14], which measures the correlation between the performance of two tools *A *and *B *as follows:(3)

Where *P*(*A *∩ *B*) is the probability of a binding site being correctly identified by the both algorithms *A *and *B*, and *P*(*X*) is the probability of a binding site being correctly identified by algorithm *X*. Clearly, *P*(*A *∩ *B*) = *P*(*A*) · *P*(*B*) if *A *and *B *are independent. Note that we restrict the log-odds ratio to the binding sites found by at least one algorithm and missed by at least one other algorithm. Unlike the Pearson correlation coefficient that may evaluate two high-performing algorithms to be highly correlated, because some binding sites may be found by both algorithms, and some others may be also missed by both algorithms, the log-odds ratio is able to capture the subtle difference in the results of two high-performing algorithms.

## Availability

The programs of MotifClick were implemented in standard C++. The source code, executables and a web server of MotifClick are freely and publicly available at: http://motifclick.uncc.edu/.

## Abbreviations

TF: transcription factor; PSSM: position-specific scoring matrices; oops: one occurrence per sequence; anr: any-number of repetitions; SSD: sum of squared distance; nCC: nucleotide-level correlation coefficient; CC: correlation coefficient; TP: true positive; FP: false positive; FN: false negative; Sn: sensitivity; Sp: specificity; PC: performance coefficient; lbSp: low bound of specificity; BDBH: bidirectional best hits; *E. coli*: *Escherichia coli*; *B. subtilis*: *Bacillus subtilis*; *S. cerevisiae*: *Saccharomyces cerevisiae*; *S. bayanus*: *Saccharomyces bayanus*; *S. castellii*: *Saccharomyces castellii*; *S. kluyveri*: *Saccharomyces kluyveri*; *S. kudriavzevii*: *Saccharomyces kudriavzevii*; *S. mikatae*: *Saccharomyces mikatae*; *S. paradoxus*: *Saccharomyces paradoxus.*

## Competing interests

The authors declare that they have no competing interests.

## Authors' contributions

ZS conceived the project. SZ designed and conducted the experiment. SL, MN, and PP helped conduct some analysis. ZS and SZ wrote the manuscript. All authors read and approved the final manuscript.

## Supplementary Material

Additional file 1 **Additional file **1**consists of two tables and 3 figures**. Table S1: Log-odds ratios of pairs of programs for predicting binding sites of eight bases with an SSD ≤ 0.06 in the synthetic datasets. Table S2: Log-odds ratios of pairs of programs for predicting binding sites of 16 bases with an SSD ≤ 0.06 in the synthetic datasets. Figure S1: Comparison of MotifClick with other three algorithms for noise tolerance. These algorithms were evaluated on three groups of synthetic datasets with sizes 400*20 (without added noise), 400*25 (with 25% added noise, and 400*30 (with 50% added noise) bases. Figure S2: Average running time of MotifClick for finding motifs of different length in different sizes of input sequence sets. The corresponding standard errors are denoted by vertical barbs. Figure S3: Evaluation of sensitivity (Sn) and specificity (Sp) of the algorithm on real datasets with different SSD values. Click here for file
