# Supplementary material for: MotifClick: prediction of cis-regulatory binding sites via merging cliques
Source: BMC Bioinformatics. 2011 Jun 16;12:238. doi: 10.1186/1471-2105-12-238 (PMC3225181; doi:10.1186/1471-2105-12-238)
Supplement: Additional file 1 — consists of two tables and 3 figures. Table S1: Log-odds ratios of pairs of programs for predicting binding sites of eight bases with an SSD ≤ 0.06 in the synthetic datasets. Table S2: Log-odds ratios of pairs of programs for predicting binding sites of 16 bases with an SSD ≤ 0.06 in the synthetic datasets. Figure S1: Comparison of MotifClick with other three algorithms for noise tolerance. These algorithms were evaluated on three groups of synthetic datasets with sizes 400*20 (without added noise), 400*25 (with 25% added noise, and 400*30 (with 50% added noise) bases. Figure S2: Average running time of MotifClick for finding motifs of different length in different sizes of input sequence sets. The corresponding standard errors are denoted by vertical barbs. Figure S3: Evaluation of sensitivity (Sn) and specificity (Sp) of the algorithm on real datasets with different SSD values. [file 1471-2105-12-238-S1.PDF]

S. Zhang *et al.*, Tables S1 and S2

Table 1. Log-odds ratios of pairs of programs for predicting binding sites of eight bases with an SSD ≤0.06 in the synthetic datasets

	BioProspector	MEME	MotifCut	Weeder
MotifClick	0.15	0.12	0.14	0.19
Weeder	0.18	0.16	0.15	
MotifCut	0.21	0.23		
MEME	0.35			

Table S2. Log-odds ratios of pairs of programs for predicting binding sites of 16 bases with an SSD ≤0.06 in the synthetic datasets

	BioProspector	MEME	MotifCut
MotifClick	0.12	0.09	0.10
MotifCut	0.17	0.19	
MEME	0.31		

S. Zhang *et al.*, Figure S1

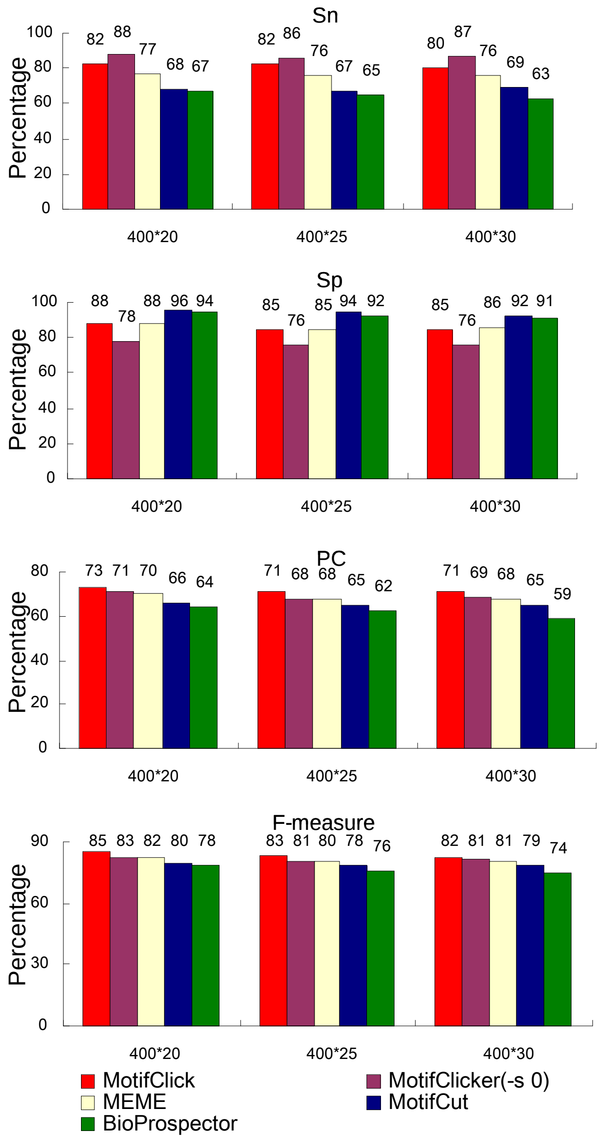

Figure S1. Comparison of MotifClick with other three algorithms for noise tolerance. These algorithms were evaluated on three groups of synthetic datasets with sizes 400*20 (without added noise), 400*25 (with 25% added noise, and 400*30 (with 50% added noise) bases.

S. Zhang *et al.*, Figure S2

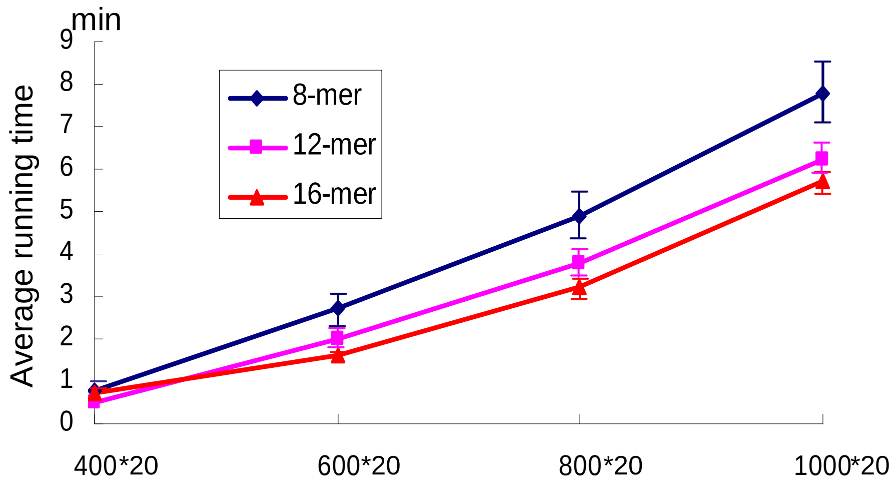

Figure S2: Average running time of MotifClick for finding motifs of different length in different sizes of input sequence sets. The corresponding standard errors are denoted by vertical barbs.

S. Zhang *et al.*, Figure S3

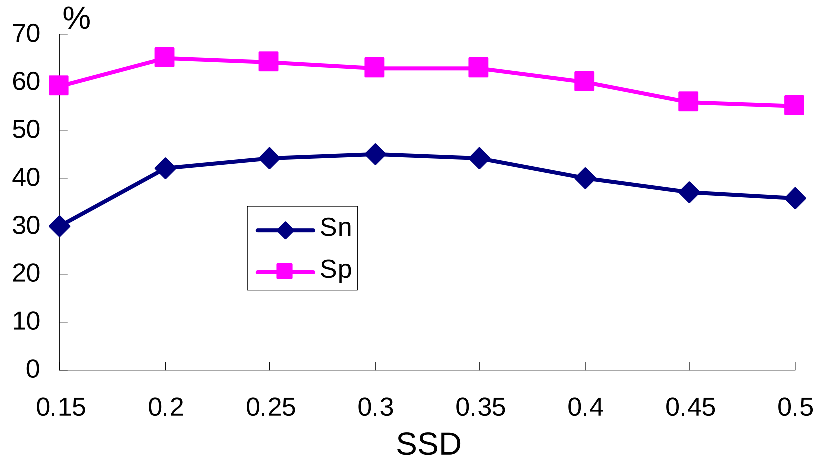

Figure S3: Evaluation of sensitivity (Sn) and specificity (Sp) of the algorithm on real datasets with different SSD values.
